# Visualizing the Genome: Experimental Approaches for Live-Cell Chromatin Imaging

**DOI:** 10.3390/cells11244086

**Published:** 2022-12-16

**Authors:** Vladimir S. Viushkov, Nikolai A. Lomov, Mikhail A. Rubtsov, Yegor S. Vassetzky

**Affiliations:** 1 Department of Molecular Biology, Faculty of Biology, Lomonosov Moscow State University, 119234 Moscow, Russia; 2 Department of Biochemistry, Center for Industrial Technologies and Entrepreneurship, I.M. Sechenov First Moscow State Medical University (Sechenov University), 119435 Moscow, Russia; 3 CNRS UMR9018, Université Paris-Saclay, Gustave Roussy, 94805 Villejuif, France; 4 Koltzov Institute of Developmental Biology, Russian Academy of Sciences, 119334 Moscow, Russia

**Keywords:** chromatin dynamics, 3D genome, chromatin visualization, FROS, ParB-INT, CRISPR imaging, TALE imaging, zinc finger imaging, live-cell imaging

## Abstract

Over the years, our vision of the genome has changed from a linear molecule to that of a complex 3D structure that follows specific patterns and possesses a hierarchical organization. Currently, genomics is becoming “four-dimensional”: our attention is increasingly focused on the study of chromatin dynamics over time, in the fourth dimension. Recent methods for visualizing the movements of chromatin loci in living cells by targeting fluorescent proteins can be divided into two groups. The first group requires the insertion of a special sequence into the locus of interest, to which proteins that recognize the sequence are recruited (e.g., FROS and ParB-INT methods). In the methods of the second approach, “programmed” proteins are targeted to the locus of interest (i.e., systems based on CRISPR/Cas, TALE, and zinc finger proteins). In the present review, we discuss these approaches, examine their strengths and weaknesses, and identify the key scientific problems that can be studied using these methods.

## 1. Introduction

**Figure 1:**
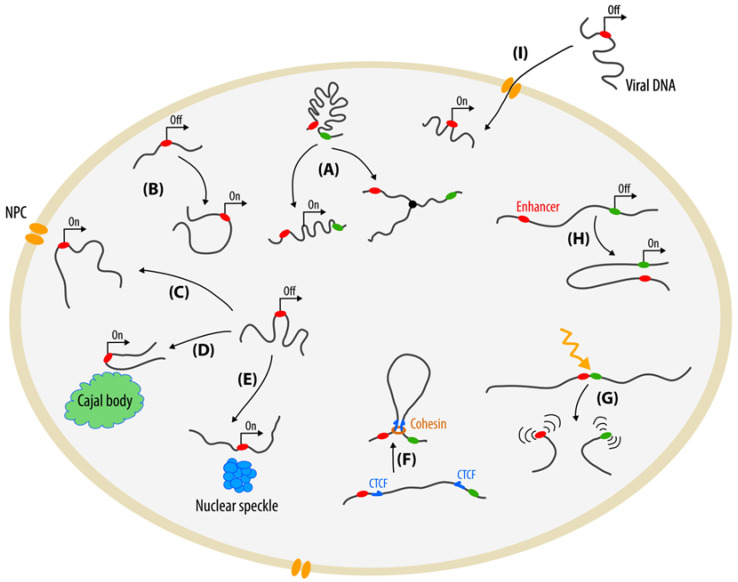
Functional mobility of chromatin loci. Green and red ellipses show arbitrary genomic loci marked only to illustrate the movements of these loci. (**A**) Changes in chromatin structure associated with transcription and replication; (**B**) Relocation of a gene from the nuclear periphery to the nuclear interior associated with transcriptional activation; (**C**) Movement of a gene to the nuclear pore complex (NPC) associated with transcriptional activation; (**D**) Movement of a gene to the Cajal body associated with transcriptional activation; (**E**) Movement of a gene to nuclear speckles associated with transcriptional activation; (**F**) Loop extrusion by cohesin complex. CTCF sites represent stop signals of extrusion; (**G**) Damage-induced chromatin dynamics; (**H**) Interaction of an enhancer and a promoter associated with transcriptional activation; (**I**) Mobility of viral DNA.

Chromatin is a highly dynamic structure (Figure 1). Movements of chromatin loci accompany all key processes in the cell nucleus, such as transcription [1] and replication [2]. Activation of transcription can lead to repositioning of a gene. Upon activation, the DHFR gene locus in CHO cells moves from the inactive compartment, the nuclear periphery, to the nuclear interior [3]. At the same time, a number of genes in *Saccharomyces cerevisiae* (*GAL1–10*, *INO1*, *HSP104*, *TSA2*, *HXK1*) move to the nuclear periphery or to the nuclear pore upon activation [4,5,6,7,8,9]. Other examples of gene relocalization associated with gene activation include the movement of the small nuclear RNA *U2* gene to the Cajal body in HeLa cells [10] and the movement of the HSP70 gene (*HSPA1A*) toward nuclear speckles in CHO cells [11].

Biochemical methods for studying genome topology, such as Hi-C, have revealed characteristic features of eukaryotic chromosome organization: the presence of topologically associated domains (TADs), loops, and compartments [12,13,14,15]. Loop formation and chromatin segregation into separate TADs in mammals proceeds by a loop extrusion mechanism [16,17,18]. Cohesin complex is believed to be an engine of loop extrusion, and CTCF protein-binding sites mark the borders of the loops. This model is in agreement with live-cell visualization experiments where the depletion of RAD21 (a component of cohesin complex) or CTCF leads to decreased chromatin looping and TAD disassembly [19,20,21]. Chromatin looping also occurs upon formation of enhancer–promoter interactions [22,23,24].

The mobility of DNA double-strand breaks (DSBs) is an example of chromatin dynamics under stress conditions [25]. In *S. cerevisiae* cells, DSBs have increased mobility compared to intact chromatin; this may facilitate the search for a homologous DNA for repair by homologous recombination [26,27]. In mammalian cells, the dynamics of DSBs is still unclear, with evidence both for [28,29,30,31] and against [32,33,34,35] an increased mobility of DSBs, which may depend on the type of DSB, the visualization system, and the cell type used. Regardless of overall mobility, the random connection of DSB ends of different chromosomes is possible and can lead to the formation of chromosomal translocations [36]. The investigation into the mechanisms of the formation of chromosomal translocations in mammals is of considerable importance since chromosomal rearrangements underlie the development of many types of cancer [37]. Viral infection may induce DNA damage and increased mobility of genomic loci [38]. This can result in chromosomal translocations. Moreover, viral nucleic acids also roam inside the cytoplasm and nucleus [39,40,41,42,43].

To elucidate the patterns and mechanisms of these and other types of chromatin movements, it is necessary to employ methods that allow direct visualization of individual loci or whole chromatin in living cells. Most methods for studying chromatin dynamics are based on fluorescence microscopy, where fluorescent molecules are attracted to the locus of interest and the movements of the fluorescent signal are subsequently tracked. Depending on the goals of a particular investigation, wide-field microscopy, confocal microscopy, or super-resolution microscopy can be used [44].

For decades, fluorescence in situ hybridization (FISH) was the principal method for studying the location of and interactions between individual chromatin regions (for a review, see [45,46]). FISH was frequently accompanied by the immunostaining of chromatin proteins. As FISH involves cell fixation steps, it is an end-point method that does not allow following dynamic events in a single cell. Although FISH can be used to conduct kinetic studies by sampling cells at different time intervals, the speed of sampling and cell fixation is limited to minutes or even hours. With sampling at such a speed, a considerable amount of information is inevitably lost, and it is impossible to study fast kinetic processes, dynamics of chromatin loci in individual cells, single particle tracking, or diffusion properties of chromatin loci. Current methods for the study of live-cell chromatin dynamics do not have the aforementioned shortcomings, as they allow researchers to visualize the location and trace the movement of certain chromatin regions in individual cells. In addition, live-cell microscopy methods are devoid of the artifacts that can occur during fixation and hybridization.

Regardless of the method used for live-cell visualization, however, it should possess a high target fluorescence intensity with a high signal-to-noise ratio (SNR), signal stability, and the possibility of long-term observations, specificity (visualization of only target sequences), non-invasiveness (i.e., the imaging method should not disturb the native dynamics of chromatin), versatility (i.e., suitable for both visualization of repetitive and unique genome sequences), convenience, and reasonable time consumption. We will consider the extent to which each method satisfies these requirements. It should be kept in mind, however, that there have been few direct experimental comparisons of different methods, and our assessments should be viewed with a critical eye. In addition, regardless of the technology employed, the specificity of target sequence labeling should be confirmed by other methods, such as FISH.

The main challenge in the development of new methods for visualizing individual loci is that the brightness of a signal from a target locus should significantly exceed the background. A high signal-to-background ratio is possible if a sufficiently large number of fluorescent molecules are assembled at the target locus. This situation is most easily achieved for loci containing repetitive sequences. Therefore, many methods have initially been tested on telomeres and centromeres prior to being adapted for the visualization of unique loci. The aggregation of many fluorescent molecules at a unique target locus requires somewhat sophisticated approaches, each of which will be considered in the corresponding sections of this review.

In the present review, we discuss a variety of methods currently available for studying chromatin dynamics in living cells, in which we focus on the critical aspects of the methods. In addition, we present the main biological discoveries made with these methods, including CRISPR imaging, techniques based on bacterial repressors and operators (e.g., fluorescent repressor-operator systems, FROS), bacterial systems of chromosome segregation (e.g., ParB-Int), zinc finger proteins (ZFPs), and transcription activator-like effectors (TALE) proteins. The aim of our review is to aid researchers in choosing a method that fits their goals, as well as to discuss the potential pitfalls of each method.

## 2. Whole Chromatin Visualization

DNA-binding dyes, such as Hoechst33342, Hoechst33258, or DRAQ5, are often used to label whole chromatin in a living cell [47,48,49]. Hoechst33342 and Hoechst33258 are cell-permeable nucleic acid stains that emit blue fluorescence upon binding with the minor groove [50]. DRAQ5 is an anthraquinone intercalator with red/far-red excitation/emission [51,52]. These dyes bind preferentially to AT-rich sequences, thus predominantly enabling visualization of heterochromatic regions [50,52]. While staining chromatin with DNA-binding dyes is crude and does not enable distinguishing different loci, a detailed pixel-by-pixel analysis of chromatin movement in a series of images by a dense optical flow reconstruction and a Bayesian inference approach makes it possible to identify chromatin regions with different diffusion properties by using a method called Hi-D [53].

When using these dyes for imaging, their possible negative effects on chromatin structure and cell viability should be considered. For example, DRAQ5 can displace histones H1 and H2B from DNA [54], lead to blockage of the cell cycle in the G2 phase [48], and induce apoptosis [52]. Hoechst family dyes also exhibit a toxic effect on cells and inhibit DNA topoisomerases, DNA helicases, and several transcription factors [50,55]. Hoechst333258 is less toxic than Hoechst33342 but has a decreased ability to penetrate cells [50]. However, the negative effects of these dyes can be minimized by selecting small dye concentrations, minimum power, and a short duration of cell irradiation [55]. SiR-Hoechst, a less toxic variant that fluoresces in the far-red region of the spectrum, is another option to consider [56].

In addition to DNA-binding dyes, the expression of histones fused to fluorescent proteins [32,47,48,54] and incorporation of fluorescent nucleotides into DNA of dividing cells [29,47] can be employed to visualize the entire chromatin. Histones fused with photoactivated proteins can be used to trace large individual chromatin regions (several micrometers in size) [32,47,48]. In addition to the standard variants, a photoactivated (photocaged) version of Hoechst33342 has recently been developed. This also enables the observation of individual chromatin regions in the nucleus [49]. Damaged chromatin can be specifically visualized by the expression of DNA damage response proteins 53BP1, NBS1 or Aprataxin fused to a fluorescent protein [34]. The aforementioned approaches, however, do not allow the study of the dynamics of specific chromatin loci, such as individual genes, centromeres, and telomeres.

Telomeres and centromeres are the easiest loci to visualize. Their dynamics can be monitored through the expression of endogenous proteins that bind to these sequences when fused with fluorescent tags. For example, TRF1-GFP is used for telomere imaging [57], and CENPB-GFP for centromere imaging [29]. However, the diversity of cellular proteins that specifically bind unique regions of the genome is low. Therefore, universal methods are needed that enable labeling of a wide variety of genomic sequences. These methods can be divided into two groups described in Section 3 and Section 4.

## 3. Tagging of Individual Chromatin Loci

The first group of methods for visualizing individual loci requires tagging a locus with an artificial DNA sequence that is inserted beforehand into the desired region of the genome. Special proteins fused with fluorescent proteins subsequently bind to this artificial DNA sequence.

### 3.1. Fluorescent Repressor-Operator Systems

**Figure 2:**
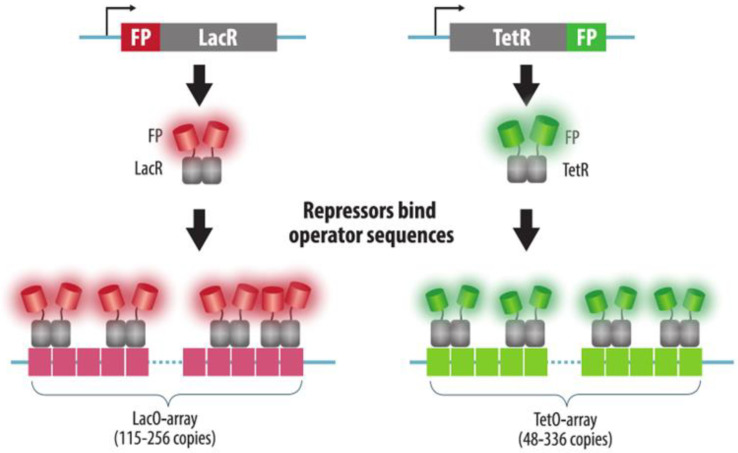
Fluorescent repressor-operator systems (FROS). Arrays of operator sequences (LacO or TetO) of variable size are integrated into the desired genomic loci. Visualization is achieved by the corresponding repressor proteins (LacR or TetR) fused to fluorescent proteins (FP) that are expressed in cells. The use of different types of FROS enables multicolor imaging of several different loci.

Fluorescent repressor-operator systems (FROS) are based on the interaction of a bacterial operator sequence and a repressor protein fused to a fluorescent protein. An array of operators is integrated into the target locus of the genome. There are two main types of FROS: one is based on the repressor and the operator of the lactose operon (LacO/LacR) proposed in 1996 [58], and the other is based on the repressor and the operator of the tetracycline operon of *Escherichia coli* (TetO/TetR) proposed a year later [59] (Figure 2). These systems enable simultaneous visualization of two different loci in cells. The FROS panel can be extended by using mutant variants of operators and appropriate repressors [60]. Repressors and operators from other microorganisms can also be employed, e.g., a visualization system based on the cuO operator and the CymR repressor from *Pseudomonas putida*) [23] or a repressor-operator pair from lambda phage (λO and λcI) [61].

As in other fluorescent technologies, it is important to achieve the formation of a bright fluorescent signal against a background created by molecules of fluorescent proteins that are not associated with the operator when using FROS. To achieve this effect, many fluorescent protein molecules must bind to the target sequence. Therefore, several tens or hundreds of copies of the operator are inserted into the target locus of the genome. In the original versions of the FROS technique, 256 copies of LacO, each 36 bp in size (with a total size of ~10 kb) [58], or 336 copies of TetO (with a total size of ~17 kb) [59], were inserted into the genome. Shorter arrays were also used, e.g., 96 (or even 48) copies of TetO [62] or 115 copies of LacO [63]. Super-resolution microscopy allows reducing this number even further to as few as six TetO repeats [64].

FROS systems have been widely used to study chromatin structure and dynamics, e.g., the spatial mobility of DSBs in murine cells [33] and yeasts [26] or chromosomal translocation formation in murine fibroblasts [36]. This system was also used to demonstrate the movement of a gene from the periphery of the nucleus to the center, upon activation of expression in CHO cells [3], and to visualize the contacts between Xic loci upon inactivation of the X chromosome [65]. With the use of the LacO/R system, large chromatin domains (several tens of millions of base pairs in size) were visualized, and their structure, replication, and transcription were studied [1,2,58,66,67]. When selecting cells with amplification of a large array of LacO, the following approach was used: cells were transfected with a construct containing LacO and the dihydrofolate reductase gene. The cells were subsequently selected with a gradually increasing concentration of methotrexate. FROS systems have also been adapted for the visualization of chromatin in plant cells, including the study of unique (non-repeating) genomic loci [68,69].

Looking ahead, we note that despite the emergence of newer technologies (primarily CRISPR imaging), FROS systems are still used in modern research. Thus, FROS was recently used in two studies to visualize the borders of chromatin loops and TADs and to study the effects of cohesin or CTCF depletion [19,21], as well as in a study of enhancer–promoter interactions [23]. Another example of the application of FROS is the identification of topological transitions, looping, and phase separation within the *IGH* locus [60]. These recent studies reflect a high degree of researcher confidence in FROS.

Despite the popularity of FROS, several studies indicate that observations using FROS should be interpreted with caution. The LacO array in mammalian cells behaves like a fragile site and the phosphorylated histone H2A.X (γH2A.X) which marks damaged chromatin accumulates within the array. Moreover, anaphase bridges containing this array are formed during cell division, and micronuclei accumulate in cells [70,71]. These effects are likely related to the replication block caused by the strong interaction between LacR and LacO. Beuzer et al. investigated in detail the effects of LacO and TetO arrays and their associated repressors on replication timing and DNA damage formation in different mammalian cell lines [71]. Expression of repressors enhanced the colocalization of PCNA with an array of operators and the degree of colocalization increased with an increase in the number of repeats in the array. In the presence of repressor proteins, replication of the array of operators persisted until the late S-phase. These effects did not occur in cells containing only an array of operators and not expressing the repressor. Thus, the aforementioned observations suggest the occurrence of a replication block caused by multiple interactions between the repressor and the operator. The replication block on the LacO array was also found in yeast cells [63]. In addition to the replication block, LacO arrays to recombine, leading to deletions in yeasts. These effects were less pronounced in cells with a lower level of expression of the Lac repressor. A decrease in repressor expression also leads to an increase in the signal-to-noise ratio [63,68]. Thus, the selection of cell clones with low repressor expression, or the use of inducible expression, is an essential step when working with FROS.

Silencing of genes located in the vicinity of the array is another potential negative effect of FROS. The insertion of arrays of LacO or TetO next to the *ADE2* reporter gene in budding yeast cells leads to its repression, but only when the corresponding repressor proteins were expressed [72]. In this case, heterochromatin proteins (SIR proteins) were attracted to the *ADE2* gene, and the gene moved to the nuclear periphery.

The propensity for recombination increases the difficulty of working with vectors that contain arrays of operators; this also impedes the integration of these constructs into the genome [62,63,68]. Inserting unique sequences between copies of the operator reduces the tendency of the array to recombine during PCR. With such an approach, it is possible to amplify the array using primers containing short homology arms to insert the array into the desired genomic locus using the CRISPR/Cas system [62].

Despite the negative effects of FROS described above, several studies found no replication disorders, problems with mitosis [2,62], or formation of heterochromatin [62]. In addition, specific functions of the chromatin into which the array of operators was embedded were not disturbed, including the interaction of the locus with the lamina or speckles [62], the timing of the X chromosome inactivation, or the choice of one of the two inactivating chromosomes [65]. Nevertheless, when working with FROS systems, it is highly desirable to conduct control experiments that demonstrate the nativeness and non-invasiveness of the system, e.g., by comparing gene expression before and after integration of an array of operators, and studying the three-dimensional structure of the locus before and after integration using C-technologies.

### 3.2. ParB-INT Systems (ANCHOR)

**Figure 3:**
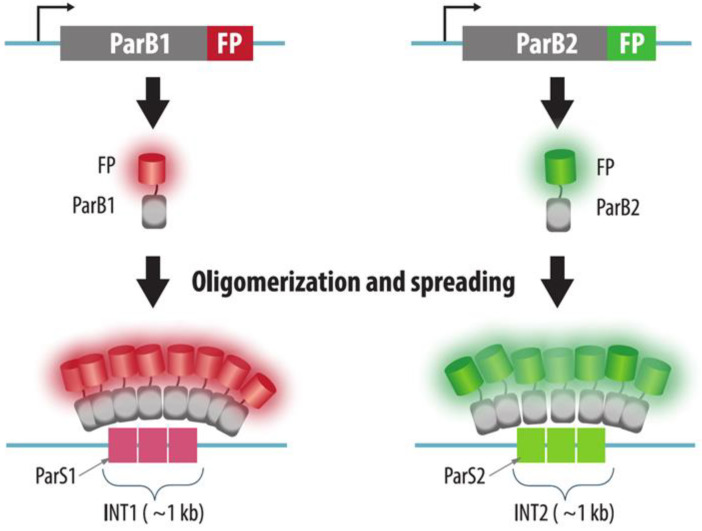
ParB-INT system. A small array of ParS sequences (INT array) is integrated into the desired genomic locus. The array is visualized by binding of ParB proteins fused to fluorescent proteins (FP) to ParS sequences, oligomerization, and spreading of ParB-FP on nearby sequences. The use of different types of ParB-INT systems enables multicolor imaging of different loci.

Another method for live-cell visualization chromatin is based on the ParABS system for the segregation of bacterial chromosomes and plasmids [73,74]. A ~1 kb cluster of ParS sites forms an array called INT which performs the function of a centromere in bacteria. This sequence is integrated into the locus to be visualized and ParB proteins fused to fluorescent proteins bind to this sequence (Figure 3).

In contrast to FROS, the increase in the brightness of the fluorescent signal in ParB-INT systems is not achieved by sequence amplification, but rather by multimerization of the ParB proteins. Several hundred ParB monomers accumulate on both the INT sequence and the surrounding DNA regions [73,74]. The authors behind the concept for this technique renamed INT as ANCH, ParB as OR, and patented some variants of this technology (ANCHOR, “NeoVirTech”). Several variants of the ANCHOR system are based on ParB and ParS from different bacterial species, enabling the simultaneous multicolor labeling of several different loci. This system may be based on ParB, ParS, or sequences of *Burkholderia cenocepacia* [73]. The origin of other variants has not been disclosed.

ParB-INT is similar to FROS, in that it is necessary to insert a specific sequence into the target locus. However, unlike FROS, ParB-INT involves the insertion of a relatively small (~1 kb) INT (ANCH) sequence [73,74], although in some cases multiple copies of the INT sequence must be inserted to enhance signal intensity [75]. Due to the small size and low repeat numbers of the inserted sequence, ParB-INT appears to be less invasive, thereby enabling the investigation of more native chromatin dynamics. Thus, interaction of ParB with INT in yeast cells did not lead to the appearance of the γH2A.X signals and did not disrupt the nucleosome assembly. Moreover, ParB interaction with INT did not significantly reduce the expression of an antibiotic resistance gene adjacent to the INT sequence [73]. When this system was employed to visualize the HCMV genome in human cells, the infection kinetics, tropism, and production of viral particles were not perturbed [39].

ParB-INT technology was first used to study the processing of DSB ends during homologous recombination in the yeast *S. cerevisiae* [73]. ANCHOR system was also used to trace the *CCND1* gene in human cells upon its activation [74]. Transcription of this gene did not disrupt OR3 binding to the ANCH3 sequence adjacent to *CCND1* promoter.

Other applications of the ParB-INT system include monitoring the replication and movement in the cell of the genomes of several types of viruses, including HCMV virus (HHV5) [39], adenovirus [40], AcMNPV baculovirus [41], and HIV-1 [42,43]. ANCHOR system was also used to investigate the interaction between the *eve* promoter and enhancer in *Drosophila melanogaster* cells [22] and to visualize the interaction of loop-forming loci (TADs) in mouse ESCs (in combination with FROS [19]). Recently, the ParB-INT system has been adapted to trace unique loci in *Arabidopsis thaliana* [76].

## 4. Methods Based on Programmable DNA-Binding Proteins

The methods described in this section are based on the use of DNA-binding proteins, the specificity of which can be “programmed” using genetic engineering. These include ZFPs, TALE proteins, and the CRISPR/Cas system widely used for genome editing (for a review, see [77,78], among others). For imaging purposes, catalytically inactive proteins fused with fluorescent proteins are employed. The drawback of this approach is that single fluorescent proteins do not create a sufficiently bright fluorescent signal, and efficient visualization is possible only for repetitive sequences. Visualization of unique loci requires the simultaneous introduction into cells of a large number of ZFPs, TALE proteins, or guide RNAs of the CRISPR/Cas system that recognize closely spaced sequences. However, there are a several ways to increase the intensity of the signals with programmable DNA-binding proteins. These approaches are discussed below.

### 4.1. Zinc Finger Imaging

**Figure 4:**
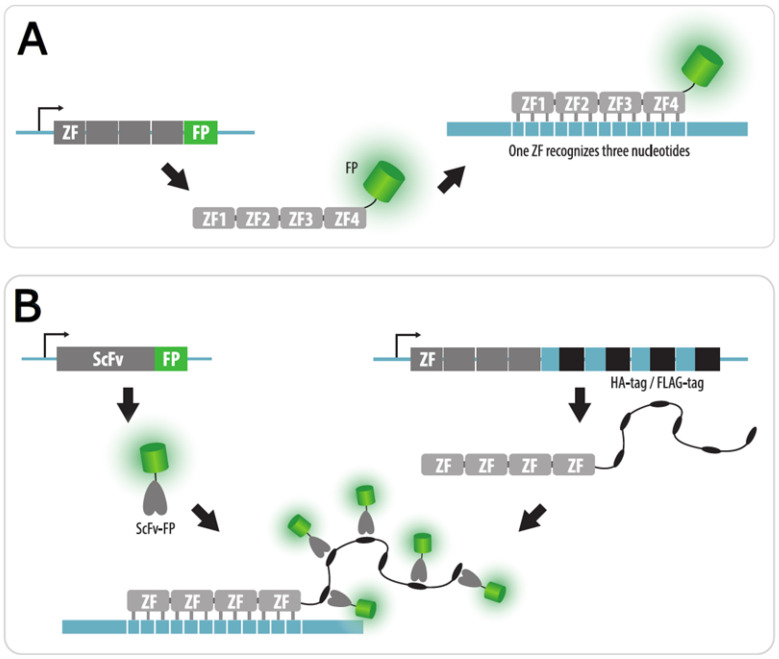
Zinc finger (ZF) imaging. (**A**) To visualize a repetitive endogenous sequence, an array of ZF modules fused to a fluorescent protein (FP) is expressed in a cell and targets this sequence. Each ZF motif recognizes three nucleotides; (**B**) Signal amplification by peptide tags. To amplify a signal, ZF modules can be fused to a cluster of peptide tags (e.g., HA-tags or FLAG-tags). These tags are recognized by a single-chain variable fragment (ScFv) fused to a fluorescent protein, which should be also expressed in cells.

An imaging technique based on fluorescent proteins with zinc finger motifs was the first method used to visualize endogenous repeats in the genome of living cells. C2H2-type zinc finger (ZF) motifs are approximately 30 amino acids in size and recognize trinucleotide sequences [79]. By combining ZF modules, DNA-binding proteins of a given specificity can be constructed (Figure 4A). In the first study, the authors visualized the pAL1 centromeric repeat (~2 × 10^3^ copies per centromere) in *Arabidopsis thaliana* root cells and the pericentromeric satellite (Major Satellite) in murine cells (1–10 × 10^3^ copies per chromosome) [80]. However, it was not possible to visualize fluorescent signals in the case of sequences with a lower number of repeats in *A. thaliana* cells (5S rRNA genes (300–350 copies), as well as a transgenic locus with 5–10 repeats). A detailed protocol for constructing ZFP and visualizing centromeres in *A. thaliana*, as well as for assessing the quantity of fluorescent proteins in the signal by calibrating with FluoSpheres, is described elsewhere [81]. According to the authors, a minimum of 200 ZFP-binding sites within the target DNA are required for successful visualization.

Casas-Delucchi et al. modified the previously described ZFP specific for the pericentromeric satellite of mouse chromosomes by adding a fragment of a nanoantibody that binds GFP with high affinity (GBP, GFP-binding protein) [82]. Such a recombinant protein can be used not only for visualization, but also for the simultaneous recruitment of various GFP-fused proteins to the centromere. In addition, by adding an estrogen receptor (ER) domain to the ZFP-GBP protein, ZFP-GBP binding to DNA can be regulated. In the absence of tamoxifen, the ER domain blocks the DNA-binding domain. After inducing the system with tamoxifen, the ER domain releases the DNA-binding domain, enabling DNA recognition.

Tol et al. used ZFP-GFP to analyze the activity of various promoters in *A. thaliana* meiocytes [83]. The *ZFP-GFP* gene was placed under the control of the promoters being investigated, and the formation of fluorescent foci on the pericentromeric chromatin was monitored. This creative, but non-obvious, approach made it possible to reduce the number of false positive results associated with cell autofluorescence, in which foci in cells are well distinguished from diffuse autofluorescence (which led to a false positive result).

The use of ZF fluorescent proteins has not become widespread, probably due to the laborious process of obtaining ZFPs of the required specificity. While protein modules that recognize almost any trinucleotide are described [84], their combinations do not always work effectively. The ZF arrays engineering performed by the assembly of the pre-characterized modules has a high failure rate [85]. Therefore, ZF-based technology is mainly substituted by modern TALE and CRISPR imaging techniques. However, studies using this approach are still encountered, since the binding of ZFPs to the target DNA is weaker than that of the complex with dCas9 and is believed to have a lower effect on the transcription and replication. For example, Liu et al. visualized unique DNA sequences by aggregating a large number of fluorescent proteins onto ZFP [86]. For this purpose, ZFPs with long peptide tags, recognized by anti-HA and anti-FLAG ScFv, fused to different fluorescent proteins were employed (Figure 4B). Cells were initially transfected with plasmids encoding ScFv-FP, and subsequently purified ZFPs with peptide tags were delivered into cells. In this study, the authors monitored the interaction of two loci, depending on the presence or absence of the cohesin complex in the cell.

### 4.2. TALE Imaging

**Figure 5:**
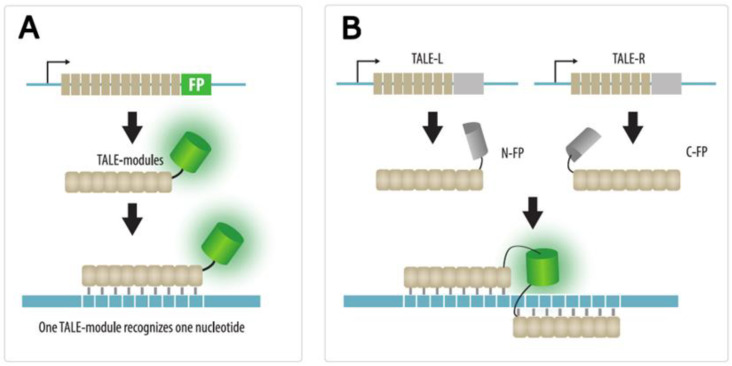
TALE imaging. (**A**) Basic concept. A fused protein consisting of TALE modules and a fluorescent protein (FP) is expressed in a cell and used to recognize and visualize endogenous genomic loci. (**B**) An approach to increase the signal-to-noise ratio by bimolecular fluorescence complementation strategy. In this case, two parts of a split fluorescent protein (N-FP and C-FP) are joined to two TALE modules that recognize nearby sequences. A functional fluorescent protein is formed only when two TALE modules interact with their target sequences, enabling two parts of an FP to dimerize.

TALE proteins from bacteria of *Xanthomonas* genus have been widely used as recognition domains for programmable nucleases. TALE proteins are a more flexible tool than ZFPs because each 34-amino acid TALE motif recognizes only one nucleotide [87,88], which facilitates the design of DNA recognition domains. To visualize a target locus, 15–20 TALE motifs are combined in one polypeptide chain and a fluorescent protein is added (Figure 5A).

The first studies using TALE proteins for imaging purposes were published in 2013 and were devoted to the visualization of highly repetitive sequences, centromeric and pericentromeric repeats, and telomeres in murine and human cells [89,90,91]. Labeling specificity was confirmed by colocalization with CENP-A and CENP-B (for centromeres) and TRF1 (for telomeres) in addition to FISH.

In these early studies, one advantage of fluorescent TALEs was already apparent: the convenience of simultaneously labeling multiple sequences in the same cell simply by using various TALE domains fused to different fluorescent proteins. The number of visualized sequences in one cell is limited only by the efficacy of the delivery of several TALE-FPs into cells and by the number of fluorescent channels in a microscope.

Visualization using TALE-FP is a relatively non-invasive approach as it does not interfere with the native chromatin structure: centromere labeling did not disturb the distribution of H3 and H3K9me3, and no mitosis defects were detected [90]. In addition, labeling telomeres with TALE-FP did not lead to their shortening or to cell cycle disorders [90]. Non-invasiveness has also been shown during in vivo visualization of centromeres using TALE-FP in developing *Drosophila* embryos [92].

TALE imaging allows to visualize telomeric and centromeric sequences for the 18S rRNA and 5S rRNA genes in *A. thaliana* cells [93]. This demonstrates higher sensitivity of TALE-FP as compared to ZFPs. Indeed, 5S rRNA genes in *A. thaliana* cells could not be observed with ZFP [80]. This advantage of TALE may be explained by an increased stability in TALE proteins binding to DNA compared to that of ZFPs [91]. When visualizing telomeres in *A. thaliana* cells with TALE-FP, the number of signals detected in the cells was always less than 20 (Arabidopsis has 10 chromosomes in a diploid cell) [93]. A similar observation (a number of signals was fewer than expected) was also made during telomere imaging in human cells [89]. This may be due to due to merging of several signals into one rather than deficiencies in TALE imaging as underestimation of the number of signals also occurs when monitoring telomeres with CRISPR imaging [94].

TALE imaging can be used as an alternative to FISH in fixed cells when it is necessary to avoid heating the sample (during DNA denaturation for FISH). Using in vitro translated TALE-FPs, Ma et al. visualized telomeres in fixed U2OS human cells [89]. Moreover, this method can be simplified if a fluorescent lysine derivative is used, which is incorporated during in vitro translation, instead of a fluorescent protein tag [89].

The signal-to-noise ratio in TALE imaging can be increased by bimolecular fluorescence complementation [95]. In this approach, a fluorescent protein, such as mVenus or mCerulean, is split into two parts, and the N- and C-fragments are fused to two different TALE proteins that recognize closely spaced sequences (Figure 5B). This significantly reduces background fluorescence and increases the signal-to-noise ratio eight-fold compared to conventional TALE-FPs.

**Figure 6:**
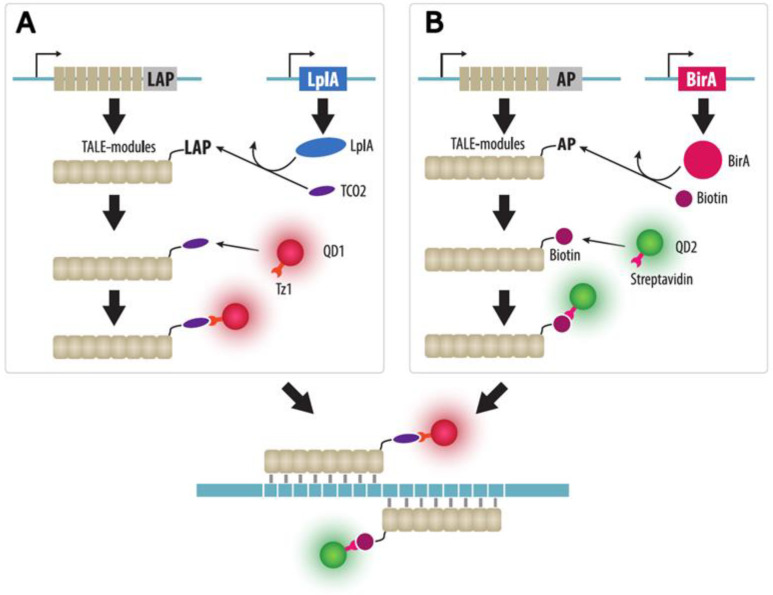
TALE imaging with quantum dots (QDs). (**A**) A first array of TALE modules fused to a LAP-tag (LplA acceptor peptide) and a LplA (lipoic acid ligase) are expressed in cells. LplA adds a trans-cyclooctene (TCO2) bridge to an LAP-tag. Tetrazine Tz1 conjugated QDs of the first color (QD1) are delivered to cells and Tz1 reacts with TCO2 via Diels-Alder cycloaddition. (**B**) A second array of TALE modules fused to an AP-tag and a BirA (biotin ligase) can also be expressed in cells. BirA adds biotin to an AP-tag, which interacts with streptavidin-conjugated QD of the second color (QD2) delivered to cells. The two arrays of TALE modules labeled with two types of QDs recognize closely located sequences.

TALE imaging is a convenient way to visualize repetitive sequences such as centromeres and telomeres. However, TALE is limited for the imaging of unique sequences by low intensity of the fluorescent signal from a single TALE-FP. Amplification of the signal by increasing the number of TALE-FPs that recognize closely spaced sequences seems to be unfeasible due to a large size of plasmids encoding TALE proteins and a low efficiency of co-transfection of cells with a large number of plasmids. However, a unique sequence in the HIV-1 provirus in human cells was visualized using TALE proteins and quantum dots (Figure 6). Quantum dots of two colors were conjugated to TALE proteins in living cells via a tetrazine-cyclooctane bridge or biotin-streptavidin using heterologously expressed enzymes [96]. The position of the target sequence (HIV-1 provirus) was determined by the colocalization of fluorescent signals from these two quantum dots, which made it possible to increase the specificity of the localization of the target sequence.

Similar to ZFPs, the construction of TALE domains to target sequences requires a complex assembly of plasmid constructs. With the advent of more user-friendly CRISPR/Cas-based systems, visualization methods using TALE proteins have become less relevant.

### 4.3. CRISPR Imaging

**Figure 7:**
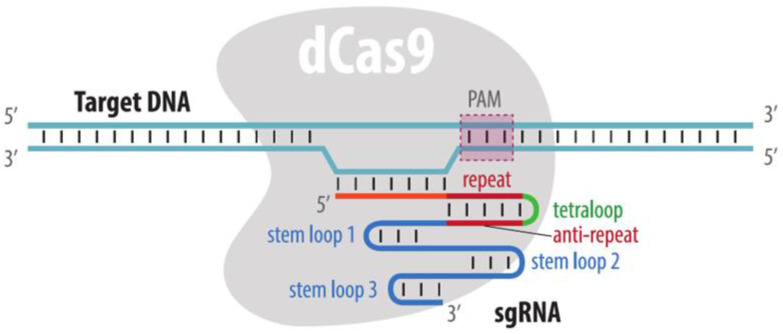
A schematic of a dCas9-sgRNA complex. dCas9, as well as Cas9, binds a sgRNA that directs this protein to a DNA sequence complementary to a guide part of sgRNA (orange). To be successfully bound by dCas9, the target DNA must contain a PAM sequence next to a sequence recognized by sgRNA. The scaffold part of a sgRNA consists of several stem loops that interact with dCas9.

CRISPR imaging is based on the use of catalytically inactive Cas9, (dead Cas9, dCas9) devoid of nuclease activity. Guide RNA (single guide RNA, sgRNA) is used to attract dCas9 to the target DNA sequence. In addition to matching the sgRNA sequence, the target DNA must contain a protospacer adjacent motif (PAM) recognized by the dCas9 protein, a requirement that imposes restrictions on the choice of target (Figure 7). Visualization of the locus recognized by the dCas9-sgRNA ribonucleoprotein complex (RNP) is carried out using either a fluorescent protein or an organic fluorophore. As in the case of genome editing, the widespread use of the CRISPR/Cas system for DNA visualization is due to the simplicity of adapting it to any target locus. To visualize the locus, one should just insert an 11–20 nucleotide recognition part of the sgRNA into the expression vector. In the following sections, we will discuss various applications and modifications of CRISPR imaging.

#### 4.3.1. Visualization by dCas9 Fused to Fluorescent Protein

The first version of CRISPR imaging appeared in 2013 [97]. EGFP-fused dCas9 protein was used to visualize repetitive sequences, telomeres, and repeats in the *MUC4* (>100 repeats in the second exon and ~90 repeats in the third intron) and *MUC1* (~140 repeats) genes in human cells. In that study, the design of the sgRNA was optimized: an A-U flip was performed in the repeat:anti-repeat duplex, as a result of which the potential termination sequence (UUUU) in the sgRNA transcript was destroyed. The repeat:anti-repeat duplex was also lengthened to increase the binding strength of sgRNA to dCas9. These modifications reduced nonspecific accumulation of dCas9 in the nucleolus and increased the signal-to-noise ratio.

**Figure 8:**
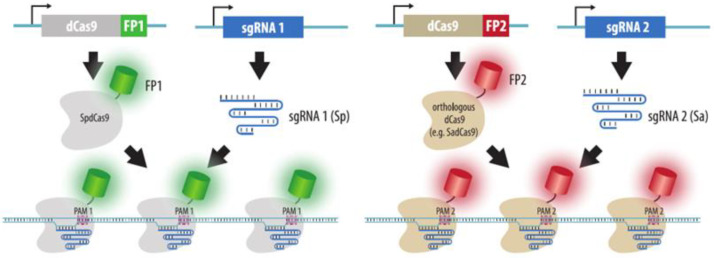
Multicolor imaging by orthologous dCas9-FP. In a basic version of CRISPR imaging, dCas9 fused to a fluorescent protein (FP) is directed to a repeated sequence by an sgRNA. The use of orthologous dCas9 proteins, for example from *Streptococcus pyogenes* (Sp) and *Staphylococcus aureus* (Sa), fused to different fluorescent proteins enables simultaneous visualization of two loci in a single cell. Note that orthologous dCas9 proteins require different PAM sequences.

Orthologous dCas9s fused to different fluorescent proteins can be used to simultaneously visualize several loci in cells (i.e., to perform multicolor imaging) (Figure 8). For these purposes, in addition to the “classical” SpdCas9 from *Streptococcus pyogenes*, dCas9 derivatives of proteins from *Neisseria meningitidis* and *Streptococcus thermophilus* [98], as well as SadCas9 from *Staphylococcus aureus* [99] can be used. These dCas9 derivatives, however, have more complex PAM sequences than those of SpdCas9 and therefore have fewer potential targets.

It is noteworthy that although CRISPR imaging was developed to study living cells, it can also be used in fixed cells as a faster and lower-temperature alternative to FISH in a method called CASFISH. HaloTag, which binds various low molecular weight fluorophores, can be used as a fluorophore fused to recombinant dCas9 [100]. Preformed dCas9 complexes with sgRNAs are quite stable and do not exchange their guides when applied to fixed cells, which makes it possible to visualize several targets simultaneously. The total protocol time, including cell fixation, hybridization, and washes, can be reduced to 15 min [100].

#### 4.3.2. Visualization of Aptamers in sgRNA Structures

**Figure 9:**
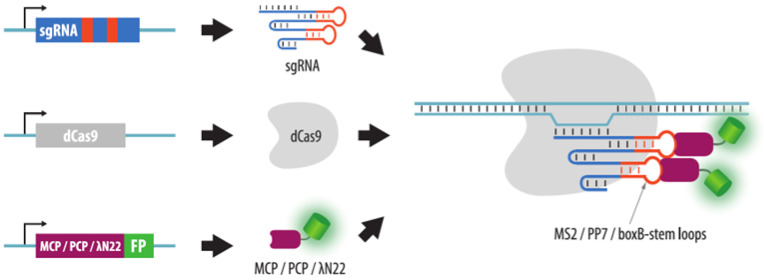
CRISPR imaging with aptamer-binding proteins. In this modification of CRISPR imaging, visualization is achieved by proteins that bind aptamers (stem loops) added to sgRNA. For this purpose, MCP, PCP or λN22 phage proteins can be used that recognize MS2, PP7 or boxB-stem loops, respectively. These proteins are fused to a fluorescent protein (FP) and are expressed in the cell, together with dCas9 and sgRNA with aptamers.

A different method of CRISPR imaging is based on the addition of aptamer modules to the sgRNA structure and expression of fluorescent derivatives of proteins that bind the aptamer sequences. Hairpins of RNA-containing bacteriophages, such as MS2 and PP7, can be used as aptamer modules. The corresponding proteins, MCP and PCP, fused with various fluorescent proteins, bind these aptamers (Figure 9). Interestingly, this imaging method enables longer in vivo observations than that with the fluorescent dCas9 as the exchange of bound and free MCP (or PCP) is faster compared to dCas9. Thus, the problem of irreversible bleaching of the fluorescent proteins is less acute [101]. To increase the affinity for aptamers, tandem derivatives of MCP and PCP can be used [101]. The efficiency of the visualization method depends on the sgRNA regions with added aptamer structures and on the number of aptamers in sgRNA [101,102]. Several variants of sgRNA structures with aptamer modules have been described, including the following: two MS2 or PP7 hairpins added to the tetraloop and a stem loop 2 sgRNA [101,103,104]; six aptamers (one in the tetraloop, another in stem loop 2, and four more at the 3’ end [102]); 14 aptamers added to the 3’ end of sgRNA [105]; 16 aptamers: 14 at the 3’ end, one in the tetraloop, and one in stem loop 2 [105] (refer to the schematic structure of sgRNA in Figure 7).

In addition to the PP7 and MS2 aptamers, BoxB hairpin, which binds to the lambda N22 peptide fused with a fluorescent protein, can be used as the third signal [104]. The combination of three basic elements (PP7, MS2, and boxB) in the sgRNA structure with the subsequent analysis of signal combinations makes it possible to visualize up to seven loci in one cell, and in addition provided the name for this approach: CRISPRainbow.

**Figure 10:**
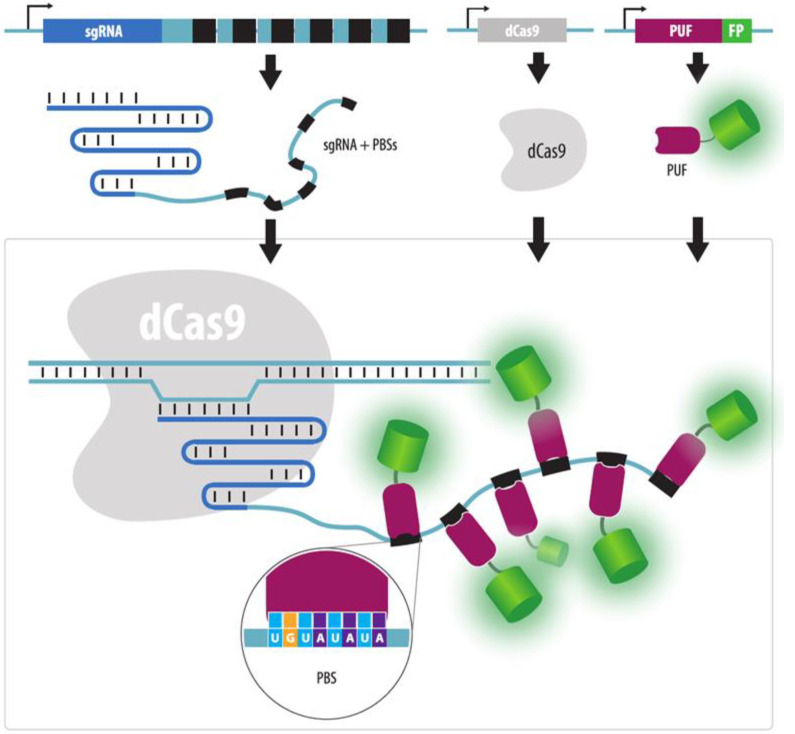
Casilio system. sgRNA is decorated by several copies of a PBS-tag consisting of eight nucleotides. PBS is bound by the PUF protein domain that contains eight motifs, with each motif recognizing one nucleotide in PBS. PUF protein fused to a fluorescent protein (FP), dCas9 and PBS-containing sgRNA are expressed in the cell. By using different PBS/PUF pairs it is possible to visualize multiple different sequences within a single cell (although different fluorescent proteins are needed).

Another tag that can be added to the 3’ end of the sgRNA is the eight-nucleotide PBS (PUF-binding site) which interacts with the PUF protein domain (Pumilio and FBF). PUF contains eight peptide motifs, each of which recognizes one nucleotide (Figure 10). It is relatively easy to program the specificity of the PUF domain by combining these protein modules [106]. Using various PBS tags attached to different sgRNAs, it is possible to simultaneously visualize numerous loci, with the so-called Casilio system [20,107]. With the use of 8 nt PBS, over 65,000 different PBS/PUF pairs can be generated. Therefore, the number of different visualized loci is determined by the capacities of the microscope: the number of fluorescent channels and the ability to distinguish combinations of several colors. A large variety of PBS/PUF pairs makes it possible both to visualize several loci in cells and to simultaneously attract, e.g., epigenetic modifiers to these loci.

**Figure 11:**
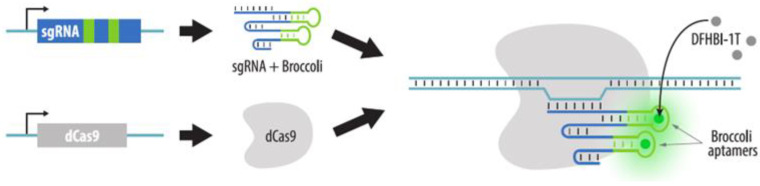
CRISPR imaging with Broccoli aptamers. dCas9 and sgRNA with Broccoli aptamers are expressed in cells. Visualization is achieved by a small molecule (DFHBI-1T) that binds Broccoli aptamers and fluoresces.

Broccoli aptamers, which are not recognized by proteins but directly bind the fluorescent ligand, can also be added to the sgRNA (Figure 11). Their ligand is the DFHBI-1T molecule, which fluoresces only when bound to the aptamer [108].

Multicolor imaging using aptamer modules has several advantages over the use of orthologous dCas9 proteins. Thus, SpdCas9, which has a short PAM (NGG), can be employed to label all loci. In addition, hairpin-binding proteins are much smaller than Cas9 proteins. Therefore, the delivery of such constructs into cells is a simpler task. It should be noted that in the absence of guides, hairpin-binding proteins and dCas9 tend to accumulate in the nucleolus [103].

Multicolor CRISPR imaging with aptamers can be used to visualize different alleles in cells [109]. In this case, the alleles must differ by more than one nucleotide since several different sgRNAs must be attracted to them for the signals to be visible.

#### 4.3.3. Visualization by dCas9 Conjugated to Organic Fluorophores

**Figure 12:**
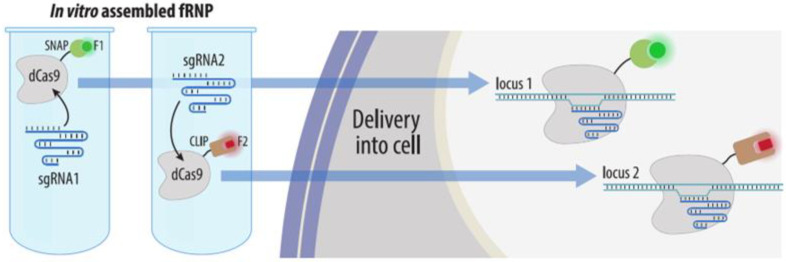
Two-color visualization by dCas9 conjugated to organic fluorophores. Recombinant dCas9 molecules fused to SNAP or CLIP tags are conjugated to organic fluorophores and form complexes with in vitro transcribed sgRNAs. These complexes are subsequently delivered into cells for visualization of target loci.

For the purposes of visualization, dCas9-gRNA RNP complexes can be formed in vitro using dCas9 labeled with various fluorescent labels. To attach these labels, SNAP and CLIP tags can be added to dCas9. Then, dCas9-SNAP and dCas9-CLIP complexes are conjugated in vitro with different fluorophores and delivered into cells (Figure 12). Due to the strength of the dCas9-gRNA complex, dCas9s do not exchange their guides in living cells, which enables the simultaneous visualization of several different loci without cross-reactivity [24].

#### 4.3.4. Visualization with gRNAs Conjugated to Organic Fluorophores

**Figure 13:**
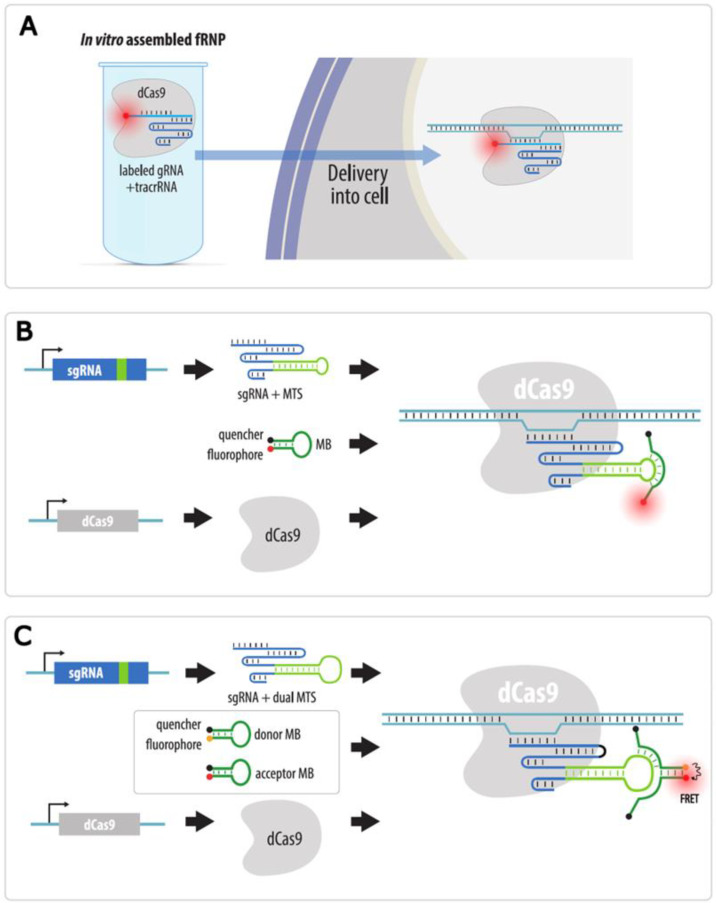
CRISPR imaging with organic fluorophores conjugated to sgRNA or to sgRNA complementary oligonucleotides. (**A**) A complex of recombinant dCas9, fluorophore-labeled gRNA, and tracrRNA is pre-assembled in vitro and delivered into cells; (**B**) MB/MTS strategy where dCas9 and sgRNA-containing MTS are expressed in cells. Molecular beacon probe containing a fluorophore and a quencher is transfected into cells. Upon binding of MB to MTS in sgRNA, the fluorophore separates from the quencher and fluoresces; (**C**) Dual FRET MB strategy. As in B, but two MBs are transfected into cells: donor MB and acceptor MB, each containing a fluorophore and a quencher. Upon binding of these MBs to MTS in sgRNA, fluorophores separate from the quenchers and become juxtaposed, enabling FRET signal emission.

Another visualization method is based on the use of organic fluorophores added during in vitro synthesis to sgRNA or to oligonucleotides complementary to sgRNA. A method called LiveFISH is based on the addition of a fluorophore to the gRNA itself, and then the RNP complex of dCas9, fluorescent gRNA, and tracrRNA is transfected into cells [110] (Figure 13A). Otherwise, sgRNA can be synthesized and labeled in vitro and then transfected into cells expressing dCas9 [111]. In a study by Wu et al., dCas9 and sgRNA were expressed in cells, and a fluorescent probe containing a fluorophore and a fluorescence quencher was transfected (molecular beacon, MB) into these cells [112]. The expressed sgRNA contained a sequence complementary to the probe (MTS, molecular beacon target sequence). When the MB-probe binds the MTS, the fluorophore and quencher move away from each other, and a signal is emitted (Figure 13B). By using different MTSs in sgRNAs and MBs fused with different fluorophores, the authors were able to simultaneously visualize telomeres and centromeres in the same cell. A modification of this method consists in using probes containing FRET pairs rather than molecular beacons and recording the FRET signal (Figure 13C). This approach further reduces background fluorescence from unbound probes and allows visualization of unique sequences using only three sgRNAs [113].

Imaging with the use of fluorescent sgRNAs or probes has several advantages over imaging using fluorescent dCas9 proteins or fluorescent hairpin-binding proteins as the brightness and stability of organic dyes exceed those of fluorescent proteins [112]. In addition, no significant modifications to dCas9 or sgRNAs that could reduce the stability of these molecules or change the dynamics of the visualized locus are required for this method. However, this approach does not allow the imaging system to be fully genetically encoded, and therefore such systems cannot be used to observe chromatin loci in whole organisms (e.g., in developing embryos).

#### 4.3.5. Strategies to Increase Brightness and SNR in CRISPR Imaging

The brightness of fluorescent signals can be a stumbling block in any imaging technique. In the case of CRISPR imaging, several strategies can be used to enhance brightness. A small increase in brightness can be achieved by adding several copies of the fluorescent protein to dCas9 (for example, three copies, as reported in [98]). However, in such a scenario, the size of the protein and its gene increases significantly. There are several ways to achieve a significant increase in signal brightness without increasing the size of the construct merged with dCas9 many times over. For example, small peptide tags can be added to dCas9, which are then bound by fluorescent proteins. Alternatively, fragments of fluorescent proteins are added to dCas9, to which the remaining parts of these fluorescent proteins bind. This method is called bimolecular fluorescence complementation, or split-FP (BiFC).

**Figure 14:**
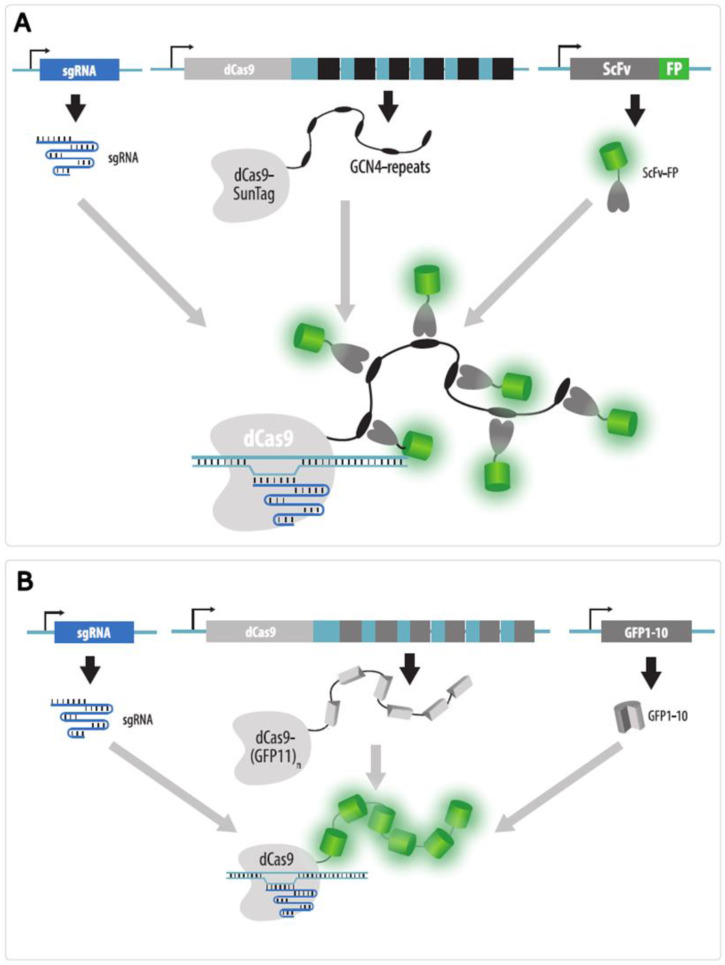
Approaches for increasing the brightness of CRISPR imaging. (**A**) SunTag technology. An array of GCN4 peptide tags is added to dCas9 expressed in cells. GCN4 repeats are recognized by ScFv fused to a fluorescent protein (FP); (**B**) Bimolecular fluorescence complementation strategy (Split-FP) in which an array of fluorescent protein fragments (e.g., GFP11) is added to dCas9 expressed in cells. A complementary part of the same fluorescent protein (e.g., GFP1–10) is expressed in cells and forms a whole fluorescent protein on dCas9. Both strategies enable the targeting of multiple copies of a fluorescent protein to dCas9 without a significant increase in the fused protein size.

SunTag technology is based on the addition of peptide tags to which several fluorescent protein monomers are simultaneously bound [114]. Several copies (up to 24 in the original study) of the GCN4 peptide are added to dCas9, and a fluorescent protein fused to a single-chain variable fragment (ScFv) that binds to this peptide is expressed in cells (Figure 14A). The signal brightness when using the SunTag technology for telomere imaging increased by 20 times compared to conventional dCas9-GFP [114]. An even greater increase in brightness (and signal-to-noise ratio) can be achieved by using a brighter fluorescent protein, such as mNeonGreen [115]. Recently, another system similar to SunTag has been developed; it is based on the interaction between a short gp41 peptide and the nanoantibody against gp41 (MoonTag, [116]). Using these two systems, it is possible to amplify the brightness of two different signals. To our knowledge, however, no studies to date using MoonTag for imaging DNA loci have been published.

In the case of the BiFC strategy, several copies of one of the fluorescent protein fragments (e.g., the GFP11 fragment in the case of Split-GFP) are added to dCas9, and the rest of the fluorescent protein (GFP1–10 fragment) is expressed in cells (Figure 14B). A functional fluorescent protein is formed only when these fragments contact each other [117,118]. When 14 copies of the GFP11 fragment were added to dCas9, the signal-to-noise ratio increased three-fold compared to the use of dCas9-GFP [117]. Since there are different split proteins (for example, Venus-N and Venus-C [119]), this method allows for the amplification of signals even with multicolor labeling.

When using fluorescent proteins that bind to aptamers added to sgRNA, signal enhancement can be achieved by increasing the number of MS2 and PP7 hairpins [120,121] or PUF-binding sites [20] in sgRNA. However, an increase in the number of hairpins does not always increase the signal brightness and may even lead to a decrease in the signal-to-noise ratio [94]. Such modifications can also reduce the stability of the sgRNA and, as a result, do not provide a substantial increase in signal intensity [121]. Ma et al. proposed a sgRNA design with eight MS2 aptamers added to the tetraloop and anti-repeat. To reduce possible recombination, point mutagenesis of aptamers was performed [120,121]. This design, which the authors called CRISPR-Sirius, was more stable and produced brighter signals than the previously reported design with 14 MS2 aptamers added to the 3’ end of the sgRNA [105].

**Figure 15:**
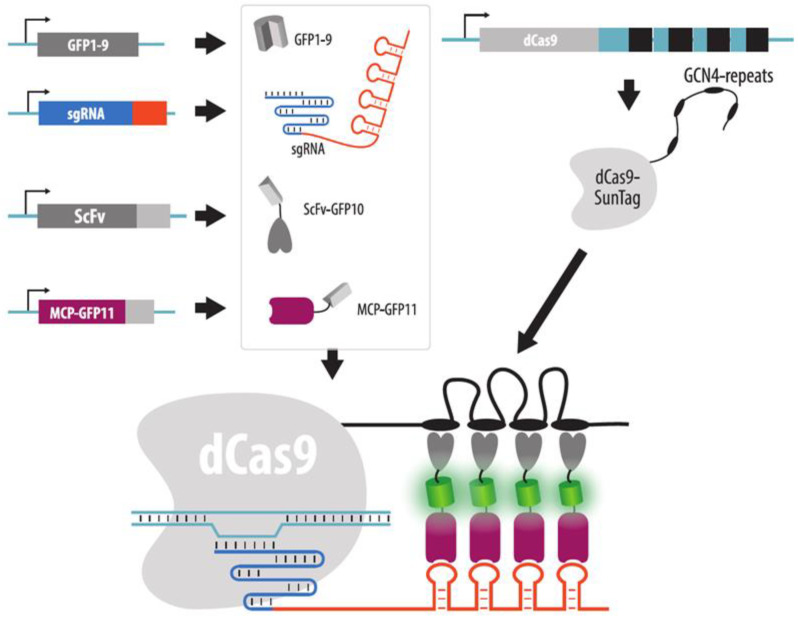
Increasing brightness and suppressing background fluorescence in CRISPR imaging by a combination of Split-FP and SunTag systems. Up to five genes should be expressed in a cell: dCas9 with GCN4 repeats (SunTag), sgRNA with MS2 stem loops, ScFv fused to a part of a FP (GFP10), MCP fused to a second part of a FP (GFP11) and the remaining part of a FP (GFP1–9). Assembly on a target locus results in the formation of a functional fluorescent protein complex.

Imaging with aptamer-binding proteins has a distinct disadvantage, however. Non-specific signals can be accumulated at the transcription sites of sgRNAs with aptameric modules. This problem was demonstrated for the Casilio system (20 copies of PBS in the sgRNA) or guides containing MS2 aptamers [119]. To combat these nonspecific signals, a strategy of bimolecular fluorescence complementation can be used. One part of the fluorescent protein (for example, Venus-C) is added to dCas9, and the other part (for example, Venus-N) is added to the aptamer-binding protein. In this case, a full-length copy of the fluorescent protein is formed only at the site where both dCas9 and sgRNA bind, which makes it possible to reduce background fluorescence and protect against nonspecific foci at the sites of sgRNA transcription. The SunTag system can also be used in this case to amplify the signal by attaching GCN4 repeats to dCas9 and expressing the corresponding ScFv fused to one half of the fluorescent protein in the cell, along with expressing the aptamer recognition protein fused to the other half of the fluorescent protein [119]. In addition to bipartite fluorescent proteins, tripartite proteins can also be used. One part of the protein (e.g., GFP10) is fused to the ScFv recognizing the GCN4 epitope (SunTag), another part (e.g., GFP11) is added to the aptamer-binding protein, and the third part is expressed in a free form (GFP1–9) [122] (Figure 15).

To increase the signal-to-noise ratio, it is sometimes necessary to select cells with a reduced expression of dCas9-FP [101] or, in the case of an aptamer strategy, cells with a low level of expression of hairpin-binding proteins [103]. A significant increase in the signal-to-noise ratio can be achieved by optimizing the structure of the sgRNA, e.g., replacing the A-U pair with the G-C pair in the repeat/anti-repeat duplex of the sgRNA [104,123].

#### 4.3.6. Visualization of Unique Loci without Repeats

Visualization of repetitive sequences using CRISPR imaging is a relatively simple procedure: for this purpose, it is sufficient to express in cells one sgRNA that recognizes a repeat element (for example, when imaging telomeres or centromeres). It is much more difficult to visualize unique sequences (such as genes) that do not contain repeats. In the first work on CRISPR imaging [97], the authors managed to visualize the first intron of the MUC4 gene using 36 sgRNAs and dCas9-EGFP. For the delivery of sgRNA genes into cells, lentiviral vectors containing 5–6 sgRNA genes were used. It should be noted that the number of signals in the cells, even when 73 guides were used, varied significantly from cell to cell, and all three signals were detected in approximately 10% of cells (the RPE cell line used has trisomy of chromosome 3 where the *MUC4* gene is located). In principle, the delivery of a large number of sgRNA genes using lentiviruses is a laborious but achievable process: Zhou et al. delivered 1124 sgRNA genes to cells to visualize the entire chromosome 9 in HeLa cells [124].

A large number of sgRNA genes can be assembled into one plasmid using the CARGO method (chimeric array of gRNA oligonucleotides) to facilitate their delivery into cells [125]. The final plasmid construct includes an array of sgRNA genes assembled by Golden Gate cloning. The basic DNA assembly blocks contain several sequences in the following order: the region encoding the second half of the recognition sequence of the sgRNA and its scaffold, the terminator, the gene promoter of the next sgRNA, and the first half of its recognition region. Using the CARGO approach, the authors were able to assemble vectors containing up to 18 sgRNA genes and, by using several of these vectors, visualize unique sequences (genes and enhancers) in mouse embryonic stem cells.

When using RNA-binding fluorescent proteins, it is possible to reduce the number of sgRNAs required for visualization by increasing the number of protein-binding repeats in the guide. Thus, Qin et al. visualized a unique locus using four guides, each containing 16 MS2 repeats [105]. Recently, Clow et al. employed the Casilio system to visualize multiple unique loci with the use of only one guide RNA per locus [20]. In this case, guide RNAs contained 15 PBS. Such approaches significantly expand the applicability of CRISPR imaging for visualizing unique sequences, and we anticipate an increase in the number of studies using this strategy in the future.

**Figure 16:**
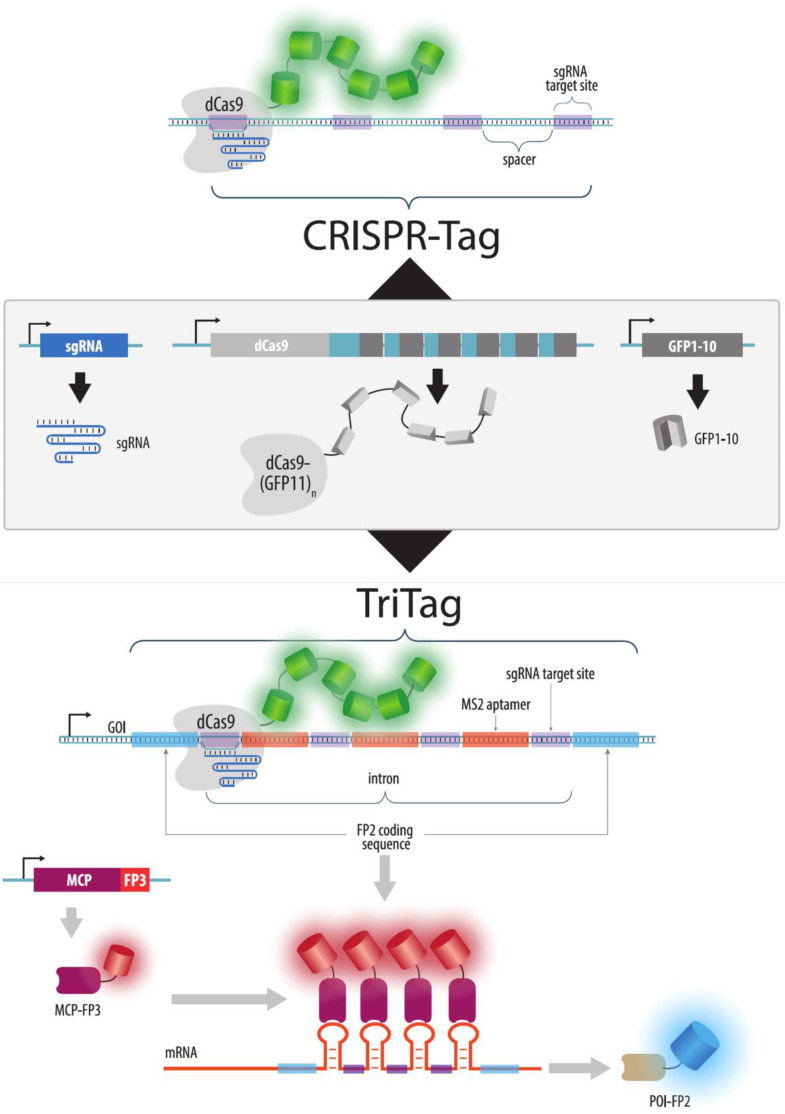
CRISPR-Tag. To visualize a locus without endogenous repeats, an artificial array (tag) of sites for high-affinity guide RNAs is integrated into the locus. This tag is visualized by a dCas9-Split-GFP strategy to increase brightness and suppress background fluorescence (**upper** part of the figure). An upgraded version of the CRISPR-Tag allows visualizing both a target gene and also imaging its RNA and protein product (**lower** part of the figure). In this case, the tag is a fluorescent protein coding sequence (FP2) added to a gene of interest (GOI). This sequence contains an intron consisting of alternating MS2-aptamers and sgRNA binding sites. The gene is visualized by dCas9-Split-GFP, its RNA by MCP fused to another FP, and the protein of interest (POI) appears to be fused to FP2.

Another strategy for visualizing unique loci using CRISPR imaging is similar to the FROS and ParB/INT systems, as it involves the integration of a special tag into the target locus. Such tags can be visualized using high-affinity sgRNAs. The published version of one particular tag [117] consists of six repeats, each containing binding sites for four sgRNAs. In this case, the BiFC strategy was applied to increase the signal-to-noise ratio: 14 GFP11 fragments were added to dCas9, and the GFP1–10 fragment was expressed in the cell to form a full-fledged GFP. Interestingly, only one sgRNA (with six sites per tag) was sufficient for visualization, albeit with a lower signal-to-noise ratio, which made it possible to reduce the tag size to 250 bp. A modified version of the tag, the TriTag, enables the simultaneous visualization of the gene, its mRNA, and the protein encoded [118]. To accomplish this process, a coding sequence for a fluorescent protein (e.g., mCherry) is added to the coding sequence of the target gene. The intron (or UTR) of fluorescent protein gene contains a CRISPR-tag and an array of 12 copies of the MS2 aptamer, to which dCas9-GFP1114x and stdMCP-tdTomato respectively bind. This strategy allows for visualization of both the DNA (labeled by dCas9) and RNA transcript (labeled by MCP), as well as a protein product of the target gene by fusing it with a fluorescent protein (Figure 16). A similar approach was previously used by Ochiai et al., but MS2 repeats themselves were employed as a DNA tag (there were 36 binding sites for three guide RNAs in such a tag) [126]; however, the brightness of the obtained signals was quite low. A disadvantage of imaging using DNA tags is the need to knock-in a tag, which can be a difficult process for some cell types and is time-consuming. Compared to FROS, however, the size of the integrated tag is lower, and it contains only a few repeats. In this respect, it is a viable alternative to the ParB-INT system.

It is worth noting that unique DNA loci can be visualized by directing dCas9 to repeats that are located near the target loci. Repeats suitable for visualization are found not only in telomeres and centromeres, but also in many other places in the genome. Appropriate repeats can be chosen, for instance, by the CRISPRbar service [121]. In addition, the recognition part of the sgRNA can be shortened to 11 nucleotides, which allows the selection of shorter, and thus more frequent, repeats as targets [104,110].

#### 4.3.7. CRISPR Imaging in Plant Cells

CRISPR imaging can be used to visualize loci not only in animal cells, but also in plant cells. Fujimoto and Matsunaga and independently Dreissig et al., visualized telomeres in tobacco cells using fluorescent dCas9 [123,127]. The number of visualized telomeres was less than 30, which is more than half the expected number of telomeres in *Nicotiana benthamiana* cells (72). The use of another strategy—sgRNAs with MS2 or PP7 aptamer modules—made it possible to increase the number of visualized telomeres in a cell to 48 and 37, respectively [94]. However, these numbers were still less than the expected number for telomeres in tobacco cells. Interestingly, attempts to obtain tobacco lines that stably expressed dCas9 [94] or dCas9-EGFP [123] for telomere visualization were unsuccessful, and there were no signals in the cells. It is possible that plants do not tolerate stable binding of dCas9 to DNA or that dCas9 harms plants in some other way, and this strategy needs to be optimized.

#### 4.3.8. CRISPR Imaging and Native Dynamics of Chromatin

In the wake of the growing popularity of CRISPR imaging, it is worth considering the “nativeness” of this technology. In most studies, CRISPR imaging did not cause any disturbances in the expression and epigenetic status of the visualized sequences. Thus, in one of the first studies of CRISPR imaging [128], dCas9-EGFP binding to the centromere did not disrupt the interaction of the CENPB protein, and dCas9-EGFP binding to telomeres did not hamper the association between the TRF2 protein and the telomeres in mESCs. Binding of dCas9-EGFP did not disrupt the expression of visualized genes: *Nanog* in mESCs [126] and *DNAJB5* in HeLa cells [124]. In agreement with this observation, the binding of dCas9-EGFP to the *Fgf5* gene enhancer in mESCs neither changed the level of the active enhancer mark, H3K27Ac nor affected the expression of the *Fgf5* gene [125]. The expression of dCas9-EGFP is not toxic for cells, as shown in experiments with transgenic mice expressing dCas9-EGFP in various tissues [129]. These mice developed normally and were fertile.

At the same time, in the first study using CRISPR imaging, dCas9 binding suppressed transcription of the *MUC1* gene in human RPE cells by 80% [97]. Athmane et al., however, called for caution in the conclusions of this study as the analyzed gene was not expressed (or was expressed at a low level) in the cell line investigated [130]. Therefore, even small absolute values of perturbation in expression could lead to a significant observed relative decrease in expression.

Recruitment of dCas9 to the *Nanog* enhancer in mESCs was found to reduce the interaction of the transcription factors *NANOG* and *KLF4* [131]. A method was subsequently developed to study the functions of individual transcription factor binding sites in mESCs based on the ability of dCas9 to block transcription factor binding [132].

Possibly, this difference in results is due to the use of different cell types, different target loci, and different levels of expression of dCas9 and sgRNAs in the studies described in this review. In any case, verification of the effect of dCas9 binding on the expression of the target gene and its epigenetic status is essential in studies using CRISPR imaging.

## 5. Resolution of Live-Cell Chromatin Imaging

We will next discuss the resolution of the described methods, i.e., a minimum distance in genomic coordinates that can be distinguished as two separate signals. The resolution of conventional fluorescent in situ hybridization in interphase nuclei ranges from tens to hundreds of thousands base pairs [46,133]. However, in recent years, FISH resolution was significantly improved by super-resolution microscopy techniques using oligopaint probes and reached 2 kb [133,134]. The resolution of live-cell imaging methods with widefield microscopy is comparable to that of the conventional FISH. Thus, using various variants of CRISPR imaging, one can distinguish loci located at a distance of about 300 kb [99], 80 kb [121] and even 70 kb [109]. Several attempts have been made to use super-resolution microscopy for live-cell chromatin imaging. Lukinavicius et al. used STED to visualize chromatin with SiR-Hoechst staining [56]. Wang et al. monitored the dynamics of telomeres and centromeres by STED and CRISPR imaging with fluorescently labeled sgRNA [111]. Single molecule localization microscopy (STROM and PALM), which requires photoswitchable fluorophores, can also be used for live imaging: Nozaki et al. used PALM microscopy to visualize chromatin with histone H2B fused to photoactivated mCherry, and added Cy3-dCTP to cell culture to visualize replicative domains in living cells [135]. Mehra et al. compensated for blurring in PALM during telomere imaging using dCas9 in a combination of PALM with wide-field microscopy, an approach called correlative conventional and super-resolution imaging [136]. FROS is also compatible with STORM, as demonstrated by the visualization of individual loci in live bacteria using the TetO/R system [64].

So far, super-resolution microscopy for chromatin imaging in living cells is represented by a small number of studies, because it is much more laborious than imaging of fixed cells. Difficulties are caused by the constant movement (at least diffusion) of the structures being imaged, as well as by the toxicity of high radiation power, which is used in super-resolution microscopy [44]. Nevertheless, super-resolution microscopy can be used for the visualization of chromatin in living cells. It is also worth noting that, for most tasks for which microscopy of living cells is applied, a very high resolution is not inevitably needed. It is always a compromise between the ability to conduct fast kinetic studies (live cell microscopy) and the study of ultra-fine structure (fixed cells).

## 6. Concluding Remarks

Methods for observing chromatin loci in living cells, which were first developed more than two decades ago, have undergone rapid changes in recent years. These new developments can be explained, on the one hand, by the discovery of proteins that recognize specific DNA sequences (ZFP, TALE, and Cas9), and on the other hand by an increase in the number of tasks that cannot be solved without such systems. Based on current trends in live-cell imaging, we assume that in the near future the principal efforts in the development of new methods will be directed toward the visualization of unique sequences. We also expect an increase in the number of studies in which these methods will be used to visualize loci not in cell cultures, but in organisms, such as developing embryos, for example.

A large arsenal of such methods will provide freedom of choice to researchers (Table 1). At the same time, it should be kept in mind that many methods (especially various modifications of CRISPR imaging) appeared in publications only once. Thus, there is no guarantee that they can be reproduced when imaging different loci in other cell types. In addition, only a small proportion of studies indicate the percentage of cells in which signals were detected, as well as the distribution of the number of signals across cells. From these observations it can be concluded that the imaging efficiency of many methods is far from 100%, and it may be necessary to test various cell clones and thoroughly looking for cells with signals during microscopy to detect cells with signals.

In this review, we did not discuss factors, such as fluorescent protein stability and phototoxicity, as they depend more on the chosen fluorescent proteins and the microscopy technique rather than on the imaging technologies themselves. However, such factors should be considered, particularly if long-term observations of cells are planned.

Finally, we would like to remind researchers who plan to use these methods to test the specificity of imaging with complementary methods, such as other live-cell chromatin imaging techniques or FISH. In addition, it is desirable to check the preservation of the expression pattern and the epigenetic status of a target sequence.

## Figures and Tables

**Table 1:** Methods for live-cell chromatin imaging.

Method	Visualization Principle	Locus Specific Imaging	Genome Editing Required	Visualization of Unique Loci without Endogenous Repeats	Advantages	Disadvantages	References to Corresponding Sections
Crude chromatin imaging with DNA-binding dyes, fluorescent histones or fluorescent nucleotides	Chromatin is stained by DNA binding dyes, by expression of fluorescent histones or by addition of fluorescent nucleotides	No	No	No	Reliable and easy to implement (compared to the other techniques)	1. Not intended to visualize specific loci	Section 2
						2. DNA-binding dyes may be toxic to cells	
FROS	Array of multiple copies of an operator sequence is integrated into the target genomic locus. Bacterial repressor protein fused to a FP is expressed in cells and binds operator sequences	Yes	Yes: insertion of an array of operators	Yes	Reliable	1. Strong binding of the repressor to the operator may cause replication block and transcription silencing	Section 3.1
						2. The need to integrate the construct into a specific locus	
						3. Difficulty in cloning repetitive constructs	
						4. A cell line must be generated for every studied locus	
ParB-INT	Bacterial INT sequence is integrated into the target genomic locus. ParB-FP is expressed in cells, binds operator sequences and oligomerizes on it	Yes	Yes: insertion of INT-sequence	Yes	A small sequence inserted into target locus. More native than FROS	1. The need to integrate the construct into a specific locus	Section 3.2
						2. A cell line must be generated for every studied locus	
ZFP	Zinc finger protein fused to a FP are expressed in cells	Yes	No	Possible by signal amplification with scFv	No need to insert a tag in the locus of interest	1. The technique is mainly applicable for highly repeated sequences	Section 4.1
						2. Complex engineering of ZFP	
TALE	TALE protein fused to a FP are expressed in cells	Yes	No	Possible by quantum dot-labeled TALE	No need to insert a tag in the locus of interest	1. The technique is mainly applicable for highly repeated sequences	Section 4.2
						2. Complex engineering of TALEs	
CRISPR: dCas9-FP	dCas9-FP and sgRNAs are expressed in cells	Yes	No *	Requires multiple sgRNA. Alternatively, CRISPR-Tag may be integrated into a locus of interest	1. No need to insert a tag in the locus of interest	1. dCas9 binding may change the expression of a target locus	Section 4.3.1
					2. Target change is easy	2. Multicolor imaging requires different dCas9 proteins, their cognate gRNAs and PAM sequences	
						3. Difficult visualization of non-repeated loci	
CRISPR: sgRNA + aptamers	dCas9, sgRNAs with aptameric modules and fluorescent proteins that bind aptamers are expressed in cells	Yes	No *	Possible with sgRNA containing multiple aptamers	1. No need to insert a tag in the locus of interest	1. Difficult visualization of non-repeated loci	Section 4.3.2
					2. Target change is easy		
					3. Easy multicolor imaging	2. Aptamer-binding proteins may accumulate in the nucleoli	
					4. High versatility and customization potential		
CRISPR: dCas9-organic fluorophore	Recombinant dCas9 conjugated to a fluorophore complexed to sgRNA are transfected into cells	Yes	No *	Difficult, requires multiple sgRNA synthesis	1. No laborious cloning	1. Difficult visualization of non-repeated loci	Section 4.3.3
					2. No need to insert a tag in the locus of interest	2. Need for transfection of fluorescent RNP prior to each experiment	
					3. Target change is easy		
					4. Organic fluorophores are bright		
CRISPR: sgRNA-organic fluorophore	Fluorescent gRNA or molecular beacon probes to gRNA are transfected into cells expressing dCas9; or fRNP of fluorescent gRNA, dCas9 and tracrRNA is pre-assembled and then transfected into cells	Yes	No *	Possible with several sgRNAs and dual-FRET molecular beacon system	1. No laborious cloning	1. Difficult visualization of non-repeated loci	Section 4.3.4
					2. No need to insert a tag in the locus of interest		
					3. Target change is easy	2. Need for transfection of fluorescent RNP, sgRNA or molecular beacons prior to each experiment	
					4. Organic fluorophores are bright		

FP—fluorescent protein(s). * Genes for dCas9, sgRNA and (if applicable) aptamer-binding proteins may be integrated into the genome to increase the efficiency. For unique loci, integration of the CRISPR-Tag can be required.

## Data Availability

Not applicable.
